# Mean reproductive traits of fungal assemblages are correlated with resource availability

**DOI:** 10.1002/ece3.1911

**Published:** 2016-01-09

**Authors:** Claus Bässler, Hans Halbwachs, Peter Karasch, Heinrich Holzer, Andreas Gminder, Lothar Krieglsteiner, Ramiro Silveyra Gonzalez, Jörg Müller, Roland Brandl

**Affiliations:** ^1^ Bavarian Forest National ParkFreyunger Str. 294481GrafenauGermany; ^2^ German Mycological SocietyDanziger Str. 2063916AmorbachGermany; ^3^ Bavarian Mycological SocietySection Bavarian ForestAblegweg 994227RabensteinGermany; ^4^ German Mycological SocietyDorfstrasse 2707751 JenaprießnitzGermany; ^5^ German Mycological SocietyKonrad‐Adenauer‐Str. 3273529 Schwäbisch GmündGermany; ^6^ Chair of Remote Sensing and Landscape Information SystemsUniversity of Freiburg79106 FreiburgGermany; ^7^ Animal EcologyDepartment of EcologyFaculty of BiologyPhilipps‐Universität Marburg35037MarburgGermany

**Keywords:** Assemblage composition, elevation gradient, fruit body, null model, sporocarp

## Abstract

Organisms have evolved a fascinating variety of strategies and organs for successful reproduction. Fruit bodies are the reproductive organ of fungi and vary considerably in size and shape among species. Our understanding of the mechanisms underlying the differences in fruit body size among species is still limited. Fruit bodies of saprotrophic fungi are smaller than those of mutualistic ectomycorrhizal fungi. If differences in fruit body size are determined by carbon acquisition, then mean reproductive traits of saprotrophic and ectomycorrhizal fungi assemblages should vary differently along gradients of resource availability as carbon acquisition seems more unpredictable and costly for saprotrophs than for ectomycorrhizal fungi. Here, we used 48 local inventories of fungal fruit bodies (plot size: 0.02 ha each) sampled along a gradient of resource availability (growing stock) across 3 years in the Bavarian Forest National Park in Germany to investigate regional and local factors that might influence the distribution of species with different reproductive traits, particularly fruit body size. As predicted, mean fruit body size of local assemblages of saprotrophic fungi was smaller than expected from the distribution of traits of the regional species pool across central and northern Europe, whereas that of ectomycorrhizal fungi did not differ from random expectation. Furthermore and also as expected, mean fruit body size of assemblages of saprotrophic fungi was significantly smaller than for assemblages of ectomycorrhizal species. However, mean fruit body sizes of not only saprotrophic species but also ectomycorrhizal species increased with resource availability, and the mean number of fruit bodies of both assemblages decreased. Our results indicate that the differences in carbon acquisition between saprotrophs and ectomycorrhizal species lead to differences in basic reproductive strategies, with implications for the breadth of their distribution. However, the differences in resource acquisition cannot explain detailed species distribution patterns at a finer, local scale based on their reproductive traits.

## Introduction

Sexual reproduction is essential for many organisms to guarantee the segregation and recombination of genes for maintaining genetic diversity (Stearns 1988). For this, organisms produce propagules (e.g., spores, seeds, eggs) in specialized organs. The production of these organs is often costly; therefore, sexual reproduction leads also to trade‐offs among traits related to reproduction (e.g., between size and number of flowers) as well as between reproductive traits and traits related to other biological functions (e.g., between reproduction and growth). Such trade‐offs and selection by the environment should lead to a reproductive syndrome characterized by a specific combination of traits that allows effective reproduction at minimal costs in a given environment. As a consequence, the reproductive syndromes of species co‐occurring in assemblages should show predictable relationships to environmental gradients (for reviews, see e.g., Clutton‐Brock 1991; Roff 1992; McGill et al. 2006).

There are two ways to approach such predictions. First, measurements of reproductive traits across species can be correlated with environmental variables measured in the habitats of the considered species. Second, the mean value of traits across all species co‐occurring within an assemblage can be analysed. Such an “assemblage approach” retrieves even subtle differences among assemblages (e.g., Gossner et al. 2013; Zeuss et al. 2014) and offers two tests of the influence of the environment on the (reproductive) trait composition of assemblages. If a (reproductive) trait affects the distribution and occurrence of species, we would expect that the mean value of the trait of an assemblage differs from that of the species pool, i.e., the set of species with the potential to colonize the considered area or habitat. Therefore, one test is to evaluate whether the mean value of a trait within assemblages differs from an expectation derived by an appropriate null model (Ulrich and Gotelli 2013). In another test, the means of (reproductive) traits across species within assemblages can be correlated with environmental variables. For example, the mean reproductive characteristics of plant assemblages depend on elevation: with increasing elevation, plant assemblages consist of more species with capsules carrying numerous tiny seeds (Pellissier et al. 2010).

Fungal spores are produced in fruit bodies. Fruit bodies show a fascinating variation in size, form, and color that rivals the morphology of flowers of angiosperms (Hibbett and Binder 2002). There is no hard evidence that this morphological variation of fruit bodies is adaptive (cf. Gould and Lewontin 1979). However, a recent cross‐species analysis showed that the size of the fruit body is correlated with the trophic lifestyle; specifically, free‐living species of saprotrophic fungi produce smaller fruit bodies than ectomycorrhizal fungi (Bässler et al. 2015).

One hypothesis argues that both the availability and distribution of resources cause this surprising difference between the two fungal guilds (Bässler et al. 2015). Both guilds need a carbon source for vegetative growth and sexual reproduction. However, the carbon sources of saprotrophic fungi and ectomycorrhizal fungi differ. Saprotrophs have to exploit a suitable substrate, which is often scarce, patchily distributed and in some cases even ephemeral (e.g., substrates of coprophilous species). Moreover, saprotrophic species need to break down the carbon of the resource with extracellular enzymes, whose production is costly (Baldrian 2008). Such limitations in the access to carbon might also explain the trade‐off between size and number of fruit bodies of saprotrophs (Bässler et al. 2015). By contrast, ectomycorrhizal fungi receive carbon from the host plant and therefore have reliable access to carbon (termed “carbon excess”, see Correa et al. 2011) in most temperate and boreal forest biomes. Thus, differences in the life history with respect to C acquisition between saprotrophs and ectomycorrhizal fungi could be interpreted within the framework of the r‐K continuum (Pianka 1970), with saprotrophs (r‐strategy) exploiting variable and unpredictable resources and ectomycorrhizal fungi adapted to a predictable resource (K‐strategy). This carbon excess for ectomycorrhizal fungi might provide degrees of freedom for reproduction have allowed the evolution of large fruit bodies and have freed these species from other environmental constraints. This hypothesis, however, assumes implicitly that large fruit bodies offer advantages, such as increased successful dispersal (reviewed in Bässler et al. 2015; see also Discussion).

To test whether the observed differences in fruit body size between the guilds is of ecological relevance, we analysed assemblages of fungi in the Bavarian Forest. This area is generally nutrient poor with a low productivity (Heurich and Neufanger 2005). If the size of fruit bodies of species of saprotrophic fungi constrains distribution, mean fruit body size of assemblages of this guild should be smaller than expected from random draws of the regional species pool (Bässler et al. 2015). Bässler and co‐workers analysed only a pooled list of species recorded along an elevation gradient. However, the deviation from the expectation should decrease along gradients of resource availability, which also exist in the investigated area. In our study presented here, we used detailed information on the distribution of species across the gradient and determined expectations by using null models that consider only the species recorded along the gradient. Such null models consider differences in the occupancy of species and therefore allow correcting for possible statistical biases. We analysed data on the composition of fungal assemblages along resource availability in the Bavarian Forest National Park (Germany) and specifically tested the following hypotheses:

1. The mean fruit body size and mean number of fruit bodies produced should differ between the two guilds across all sites. In particular, the mean fruit body size of saprotrophs should be smaller than that of ectomycorrhizal fungi and the mean number of fruit bodies produced by saprotrophs should be larger.
2. Both the mean fruit body size and the number of fruit bodies of saprotrophic assemblages should increase with increasing resource availability, but those of ectomycorrhizal fungi should not.
3. The mean fruit body size of assemblages of ectomycorrhizal fungi should not deviate from the expectation derived from the regional species pool, but that of saprotrophic fungi should be smaller than expected and the deviation should decrease with increasing resource availability.

## Material and Methods

### Fungal and environmental data

**Figure 1:**
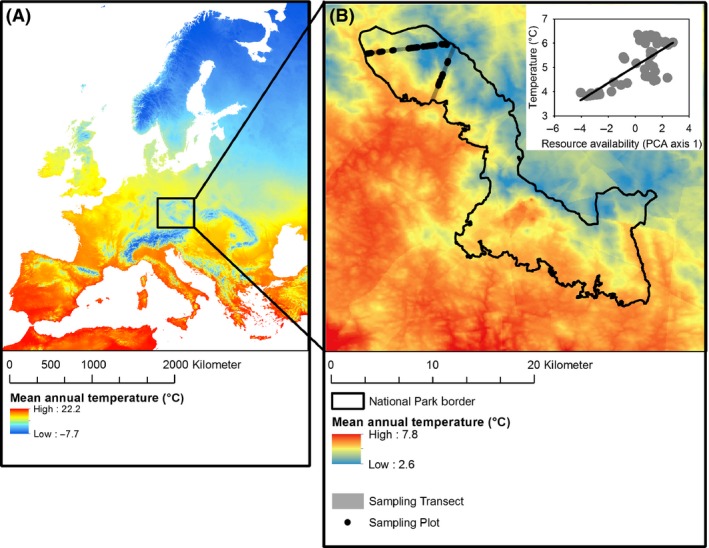
(A) Map showing the position of the study area within Europe. Colors indicate the mean annual temperature (derived from WorldClim database, www.wordclim.org). (B) Study area (Bavarian Forest National Park) showing the sampling transects (gray lines) and study plots (black dots; see Bässler et al. 2008 for details on the study design). Colors reflect mean annual temperature (1988–2007) for the study area. Inset shows the relationship between mean annual temperature and resource availability. Note that the temperatures shown in the map are outputs from a smoothing algorithm and the values may differ somewhat from the measured values of a particular site.

To analyse patterns of reproductive traits of assemblages, we sampled fungi across a resource availability gradient within the Bavarian Forest National Park in south‐eastern Germany (48°54′N, 13°29′E). The Bavarian Forest lies in the south‐western part of the Bohemian Massif, which is formed of granite and gneiss. Acidic sand and loamy soils prevail. Elevations range between 650 and 1350 m a.s.l. At 650 to 1150 m a.s.l., forests are dominated by Norway Spruce (*Picea abies*) admixed with European Beech (*Fagus sylvatica*) and Silver Fir (*Abies alba*). Above this elevation, forests are dominated by Norway Spruce and Mountain Ash (*Sorbus aucuparia*). This area is characterized by harsh climatic conditions (Walentowski et al. 2004; see also Fig. 1). At higher elevations, mean annual temperature regularly drops below 3.5°C. As a result, net primary production decreases with elevation; the growing stock of the living stand decreases from approximately 350 to 150 m^3^ ha^−1^ (Heurich and Neufanger 2005).

From 2009 to 2011, we sampled soil‐related (terricolous) macrofungi from 48 circular plots covering the available elevational gradient in stands dominated by mature spruce (Fig. 1). Plots had an area of 200 m^2^ and were surveyed weekly between June and November, i.e., during the main period of fruit body production in the study region. We counted fruit bodies at the species level and removed all fruit bodies from the plots after each survey. It has been shown that removing fruit bodies from an area has no effect on the future production of the species (Egli et al. 2006). During these field studies, 259 species were recorded. For all recorded species, we extracted from published records the trophic strategy of the species (Rinaldi et al. 2008; Tedersoo et al. 2010; Comandini et al. 2012) and the mean cap diameter d of mature fruit bodies (Knudsen and Vesterhold 2008). The size of the fruit bodies of each species was estimated as d². This index is a reliable measure of the biomass of macrofungal fruit bodies, even though it is measured in mm² (Tóth and Feest 2007; Bässler et al. 2015). From our field data, we estimated the total number of fruit bodies produced by each species by summing up the number of fruit bodies produced by each species in each year and dividing each sum by the number of plots on which we recorded that species.

For an estimate of the traits that one can expect in the regional species pool, we used a published database of fruit body size occurring in central and northern Europe (see Bässler et al. 2015 for a detailed description). This database consists of ~600 saprotrophic and ectomycorrhizal terricolous Agaricomycetes (Agaricales, Russulales, and Boletales) across 91 genera. The taxa were randomly selected on the basis of page numbers in the *Funga Nordica* (Knudsen and Vesterhold 2008) to approximately represent the proportional number of species within genera and sections of the species described in this source.

According to our hypotheses, both mean fruit body size and the number of fruit bodies of saprotrophic assemblages should be correlated with resource availability. Saprotrophic fungi rely on dead organic matter as a carbon resource for growth and reproduction. The amount of organic matter and hence resource availability for saprotrophic fungi is closely correlated with net primary productivity (Carlile et al. 2001b). However, direct estimates of net primary productivity for our area are not available. Nevertheless, net primary productivity is correlated with aboveground biomass, which is estimated by the volume of growing stock of forests (m^3^ ha^−1^) (Kimmins 2002). We therefore measured the following growing stock variables on the study plots: number of trees, volume (m^3^), basal area (m^2^), and mean diameter at breast height (DBH; m). To estimate these variables, we used full‐wave LiDAR data from airborne laser scanning (Riegel LMS Q‐560 system at a point density of 25 points m^−2^) within circular subplots of 0.1 ha at the centre of the plots used for sampling fungi. For final analysis, all values were expressed on a 1 ha basis. Laser scanning data allow discrimination between broad‐leaved and coniferous tree species and derivation of measures such as the height (m) and DBH (m) of each tree from the laser point clouds (Yao et al. 2012). From the height and DBH measures, the volume (m³) of each tree within a sample plot was calculated using indices that consider the decrease in diameter with tree height according to Heurich (2008). The algorithms used to derive these variables were developed and calibrated within our study area (for more details see Heurich 2008; Yao et al. 2012). We also visually estimated the vegetation cover on each plot (0.02 ha): % cover of shrub layer (>1–5 m), lower tree layer (>5–15 m), and upper tree layer (>15 m). We considered the age of the stand in a plot based on tree ring analysis of forest inventories (Heurich and Neufanger 2005). Variables such as growing stock, mean DBH and canopy cover are often closely correlated. We therefore subjected all variables to a principle component analysis (PCA) based on the correlation matrix. The first component accounted for 52% of the total variance; volume of the growing stock (m^3^ ha^−1^) had the highest correlation with this component (*r *=* *0.96). Basal area, mean DBH and upper tree layer were also closely correlated with the first component (*r *=* *0.90). The second component accounted for 17% of the variability; the lower tree layer and forest age showed the highest loadings on this component. We therefore used the scores of the first component as a measure of resource availability (large scores indicate a high level of resource availability) and the second component as a covariate representing forest stand succession (large scores indicate old forest stands; note that 88% of all plots are characterized by stands with a mean age of ≥ 70 years).

### Statistical analyses

We only considered species in our analysis that occurred on at least four plots to obtain a reliable measure of the mean number of fruit bodies for each species. Before we calculated the mean trait values of the assemblages, fruit body size and number of fruit bodies of each species were log_10_ transformed. In all subsequent analyses, we treated the sampling year (2009, 2010, and 2011) as a factor to account for variability among years.

For comparison of the mean trait values of local assemblages with the regional (central and northern Europe) species pool, we divided the data set according to the trophic guilds. Subsequently, we randomly selected the same number of species as observed for each assemblage from each pool and calculated the mean fruit body size across species in that random draw. We repeated this procedure 100 times for each plot and calculated the standardized effect size (SES) by subtracting the expected mean from the observed mean and dividing the difference by the standard deviation across the random draws for each plot. Values < −2 and >2 indicate significant deviations from the expectation. For the number of produced fruit bodies, only data for the local data set were available. This test was therefore only possible for fruit body size. Note that this null model simply tests whether the species recorded in the investigated area produce fruit bodies that differ in size from what one would have expected from the fruit body size of species occurring across Europe. This null model ignores factors that could influence the probability that a species colonizes a particular area (e.g., regional abundance).

The use of mean values across species and correlation of these values with other variables extracted from the matrix of species occurrences (e.g., species richness) might lead to spurious correlations (Zeleny and Schaffers 2012). The use of variables not extracted from the species‐by‐site matrix also could lead to spurious results because we expect that species richness co‐varies with the resource gradient. Therefore, we additionally used a null model that randomizes species occurrence across sites but fixes both marginal sums for sites (i.e., species richness of sites) and marginal sums for species (i.e., occupancy of sites across the 48 plots). As described above, we first divided the data set according to the trophic guilds and calculated the observed mean size and number of fruit bodies for each plot. We then randomized the community data matrix 100 times for each guild with the independent swap algorithm (Gotelli 2000). We calculated for each randomized community matrix the mean fruit body size as well as the mean number of produced fruit bodies of the randomized assemblages. Finally, we calculated the standardized effect size by subtracting the expected mean (mean across all randomizations) from the observed mean and dividing the difference by the standard deviation across the randomizations for each plot (see above). Note also that the use of separate null models for the two guilds for calculating effect sizes also removes differences in the mean fruit body size between guilds (see Table 2). By contrast, if our null model with regional data produced deviations from the expectation only for saprotrophs, we would expect differences in effect sizes between the two guilds.

We used the (raw) mean fruit body size and mean number of fruit bodies as well as the standardized effect sizes to test for the influence of resource availability (first principle component, see above) on the reproductive characteristics of the fungal assemblages by applying linear mixed‐effects models using the add‐on package nlme in R (R Development Core Team, 2015). In these models, we considered both the forest stand succession (second principle component, see above) and the factor sampling year as covariates. This enabled us on one hand to quantify the relative importance of resource availability relative to each covariate, and on the other hand to assess the variability of the reproductive traits of the different fungal guilds among years. We found no significant three‐way interaction between guild, resource availability and year (results not shown). Within all models, variance was weighted based on the guild using the *varIdent* function (form = ~1|guild) to account for within‐group heteroscedasticity (Pinheiro and Bates 2000). We furthermore used the plot as a random factor to account for repeated measurements. For all comparisons among the models, we used standardized effect sizes of the parameter estimates using an expected mean of 0 (z‐values = estimates divided by the respective standard error; see Bring 1994).

## Results

In the three years of our sampling, we collected 172,176 saprotrophic fruit bodies representing 100 species, and 24,435 ectomycorrhizal fruit bodies representing 150 species (for descriptive data, see Table 1).

**Table 1:** Number of species and number of fruit bodies of mutualistic ectomycorrhizal (ECM) and saprotrophic (ST) fungal species and mean values across 48 plots sampled in the years 2009, 2010 and 2011

	2009		2010		2011	
	ECM	ST	ECM	ST	ECM	ST
All plots						
Number of species	93	54	104	70	122	72
Number of fruit bodies	9627	46,340	6211	34,365	8597	91,471
Mean across plots (0.02 ha)						
Number of species	12.4	8.0	11.0	10.2	14.5	9.9
Min number of species	5	2	2	4	4	5
Max number of species	26	14	22	25	29	21
Number of fruit bodies	2001	965	129	716	179	1906
Min number of fruit bodies	18	6	3	9	21	10
Max number of fruit bodies	1325	5583	830	2269	725	11,490

**Figure 2:**
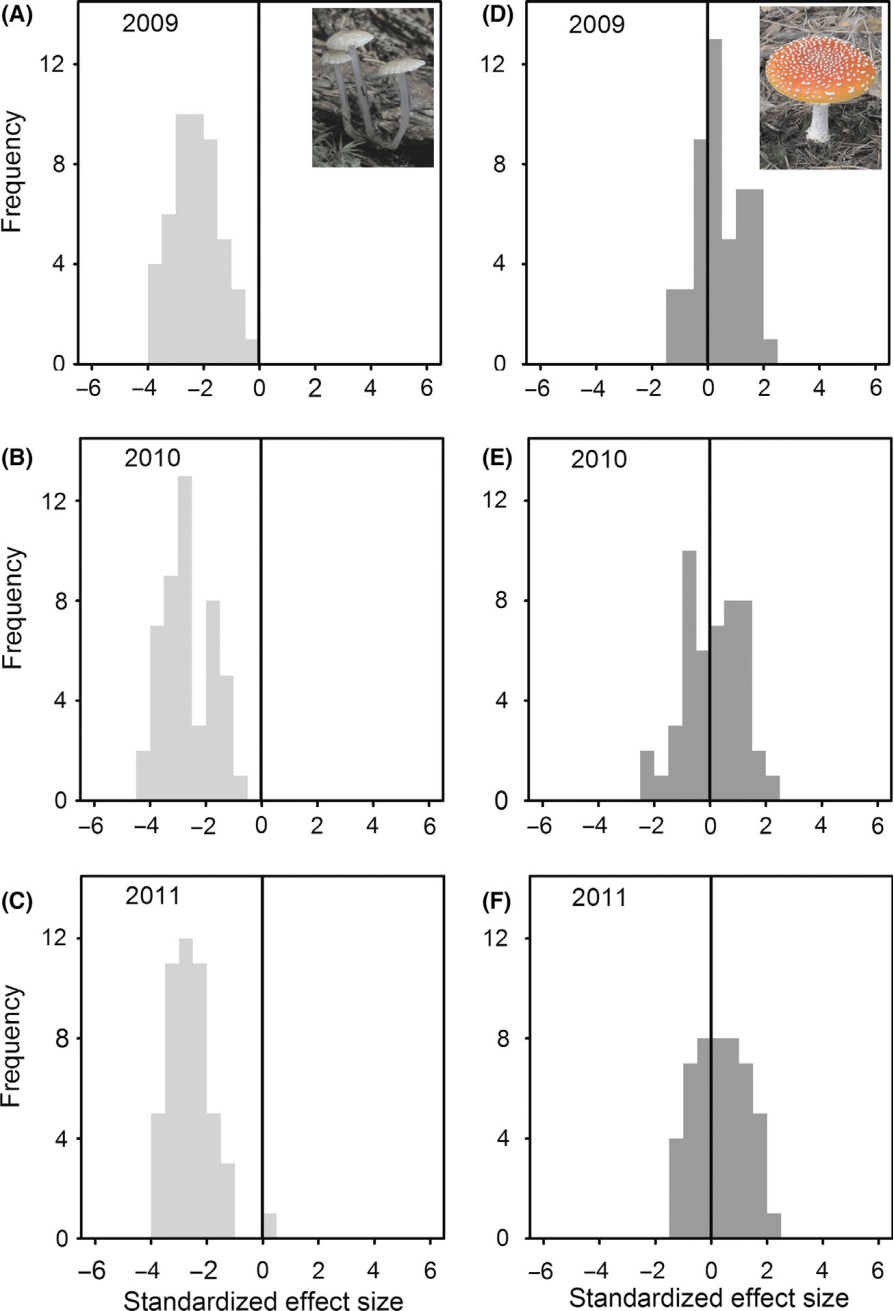
Histograms of the standardized effect sizes for each sampling year (2009, 2010, 2011) calculated from the comparison of the mean fruit body size of local assemblages (Bavarian Forest) with that of a regional species pool (central and northern Europe) of (A–C) saprotrophic fungi and (D–F) ectomycorrhizal fungi. Note that for all saprotrophic assemblages, standardized effect sizes were in most cases less than −2 (clear deviation from the regional pool), whereas for ectomycorrhizal assemblages, the effect sizes fall between −2 and 2 (no deviation from the pool). Insets in the upper panel represent typical species of the two guilds (a, *Mycena rorida* growing on a twig; b, the infamous ectomycorrhizal species *Amanita muscaria*).

The means of fruit body size and number of produced fruit bodies of the two guilds clearly differed. On average, the saprotrophs co‐occurring on a site produced smaller but more fruit bodies than the ectomycorrhizal species (Table 2). Furthermore, analysis of the effect size calculated using random draws from the regional data set demonstrated clear differences between the two guilds, which indicated that the two guilds differ in their deviation from the regional expectation. This is also underlined by our finding that standard effect sizes of mean fruit body size were less than −2 for most of the assemblages of saprotrophic fungi, whereas effect sizes of mean fruit body size of ectomycorrhizal fungi species on most plots were between −2 and 2 (Fig. 2). These results were consistent across years (Fig. 2).

**Table 2:** Results of linear mixed‐effects models to test the importance of resource availability and age of the stand considering the three sampling years (2009, 2010, 2011) on reproductive characteristics of saprotrophic (ST) and mutualistic ectomycorrhizal (ECM) fungi. Variance was weighted within the models based on the guild to account for within‐group heteroscedasticity. Plots were treated as random factors to account for repeated measurement. For all comparisons among the models, we used standardized effect sizes (SES) of the parameter estimates using an expected mean of 0 (*z*‐values = estimates divided by the respective standard error). The reference group for the test of differences (between guilds and sampling years) is indicated in italics. Significant effect sizes are in bold (*P < 0.05, **P < 0.01, ***P < 0.001). Significant differences (interaction) between the guilds are shaded gray

Reference	Guild	2009–2010		2009–2011		2010–2011		Resource availability		Age		Adj. *R*²	
	*ST*	*2009*		*2009*		*2010*							
		ECM	ST	ECM	ST	ECM	ST	ECM	ST	ECM	ST	ECM	ST
Mean fruit body size	−39.8***	−2.69**	−1.29	−0.69	−0.18	2.00*	1.11	4.00***	5.24***	−1.99*	−3.18**	0.21	0.27
Mean number of fruit bodies	19.11***	1.04	−1.81	−0.50	−2.11*	−1.54	−0.30	−4.19***	−5.61***	3.02**	2.14*	0.25	0.24
SES fruit body size regional pool	−19.05***	−2.15*	−3.37**	−0.77	−2.52*	1.39	0.85	4.56***	4.96***	−2.78*	−3.39**	0.22	0.31
SES fruit body size local pool	−0.14	−1.83	−1.74	−0.87	−0.84	0.96	0.90	4.27***	5.43***	−2.29*	−3.42***	0.20	0.30
SES number fruit bodies local pool	0.34	−0.04	−1.09	−0.25	−0.78	−0.21	0.31	−4.10***	−5.94***	3.43***	2.36*	0.23	0.30

**Figure 3:**
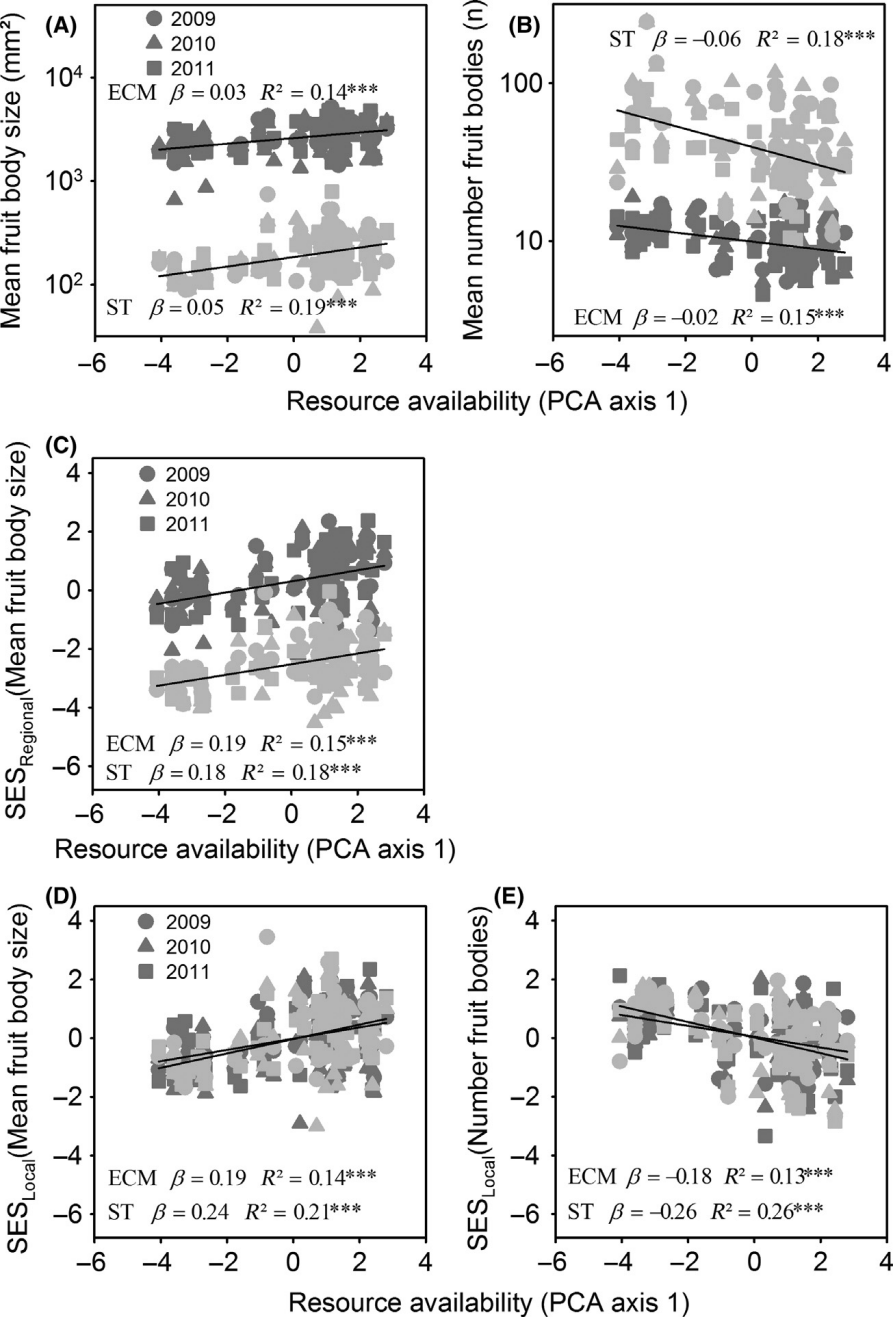
Raw scatterplots and linear regressions of (A) mean fruit body size, (B) mean number of fruit bodies, (C) standardized effect sizes (SES) of mean fruit body size (regional species pool), (D) SES of mean fruit body size (local species pool) and (E) SES of mean number of fruit bodies (local species pool) compared to resource availability (first axis of the PCA; see Material and Methods for details). Light gray symbols: saprotrophic fungi; dark gray symbols: ectomycorrhizal fungi. Different symbols represent different sampling years. Slopes (*β*) and adjusted *R*² from univariate linear mixed effect regression models that account for repeated measurement are given.

For all models and of all predictors, resource availability showed the largest effect sizes on our metrics describing both the mean fruit body sizes and the mean number of fruit bodies produced by species across sites. For both guilds, mean fruit body size increased with resource availability, and the mean number of produced fruit bodies decreased (Table 2, Fig. 3A,B). When we used the raw means, however, the slope between the two guilds differed (Table 2). This difference in the slope disappeared when effect sizes considered (Table 2). We were not able to exclude the possibility that this interaction is a result of a statistical bias (see Zeleny and Schaffers 2012); therefore, we will ignore this interaction in our discussions. Note also that for the null model with local data, most effect sizes fell within the range of −2 to 2 (Fig. 3D,E), and therefore individual effect sizes were in most cases not significant. Nevertheless, the overall trend of effect sizes across sites with respect to resource availability showed a clear pattern (Fig. 3D,E).

Effects of the age of the stand were weaker than effects of resource availability (Table 2). Nevertheless, for both guilds, we found a significant negative relationship between age and all metrics of the mean fruit body size and a positive relationship between age and the metrics of mean number of produced fruit bodies (Table 2). No model revealed a significant three‐way interaction between guild, resource availability and year (results not shown). Therefore, the basic patterns between guilds are consistent across years.

## Discussion

Our comparison of species or a species list across an entire region showed that saprotrophs not only produce smaller fruit bodies than ectomycorrhizal species, but that this difference also holds for local assemblages and is consistent across years. Furthermore, in local assemblages, species of saprotrophic fungi with small fruit body sizes co‐occur more often than expected from the regional species pool, whereas no differences were found for members of the ectomycorrhizal guild. This pattern was consistent across years and therefore supports, with some more statistical sophistication, the results of Bässler et al. 2015).

These basic results are consistent with the expectation that the different strategy of carbon acquisition of the two guilds might lead to different reproductive strategies, which in turn affects the distribution of species. If we assume that there is an upper limit to the resources a species can invest into reproduction, each species is faced with the problem of either investing in large fruit bodies or increasing the number of (small) fruit bodies. Species with small fruit bodies have the option of a finer‐grained response in reproductive investment than species with large fruit bodies. Small fruit bodies mature more quickly than large fruit bodies, which might be especially important for saprotrophic fungi (Haard and Kramer 1970). Therefore, species with small fruit bodies are able to adjust reproductive investment flexibly to specific local conditions and at the same time reduce the risk of perishing before sporulation. Our results can be understood in terms of the concept of r‐ and K‐strategies (e.g., Pianka 1970; Grime 1988). Saprotrophs follow the r‐strategy in order to exploit an unpredictable resource, whereas mutualistic ectomycorrhizal fungi follow the K‐strategy because their carbon source is more reliable (Boucher et al. 1982; Boucher 1985).

However, by following an assemblage approach and considering a gradient of resource availability within the Bavarian Forest, our study provided further insights into the structure of fungal assemblages with respect to traits involved in sexual reproduction. Across plots, all metrics that characterize the mean trait values of assemblages clearly responded to resource availability. However, in contrast to our expectation, mean values of reproductive traits of both guilds responded similarly to the local gradient of resource availability. In both guilds, the mean fruit body size increased with resource availability but the mean number of fruit bodies decreased. Furthermore, by considering the variability among years, we showed that these patterns were robust across years. However, our insights into the possible processes influencing resource acquisition, reproductive traits and distribution are not sufficient to understand all the patterns found in our study, and we acknowledge that both a carbon‐centred approach and the application of the r‐K continuum are oversimplifications.

Contrary to our expectation, assemblages of both guilds were composed of species that on average produce larger fruit bodies in highly productive environments. This leads to the conclusion that large fruit bodies should have some advantages irrespective of the lifestyle. We list five possible advantages. (1) A large fruit body can generally produce more spores than a small fruit body as fruit body size is correlated with the hymenial surface (Bässler et al. 2015) and most probably also to longevity (Richardson 1970; Kramer 1982). Fruit bodies sporulate as long as they remain vital, and therefore the number of produced spores increases as the fruit body matures (Haard and Kramer 1970; McKnight 1990; Moore et al. 2008). Hence, fungi with large fruit bodies might be less dispersal limited, which can become a major factor for both the distribution of species and species diversity (Peay et al. 2010). (2) Fruit body size of ectomycorrhizal fungi is correlated with spore size (Bässler et al. 2015). Large spores, like large seeds, might offer advantages during establishment at a site by allowing prolonged dormancy (Halbwachs and Bässler 2015). (3) Spores released from larger and therefore taller species will more easily leave the boundary layer of still air and disperse farther than spores of shorter species (Galante et al. 2011). (4) A large fruit body has a lower surface‐to‐volume ratio; this might increase protection against desiccation and pathogens, which generally seems to be critical for sporulation in agarics (Buller 1909; Clémençon 1997). (5) A large fleshy trama and stipe might act as a defence against invertebrates that feed on fruit bodies. Furthermore, large fruit bodies might attract animal dispersal vectors (Bunyard 2007).

The fact that the mean fruit body size of local saprotrophic assemblages was smaller than expected from the regional species pool suggests that saprotrophs occurring in the investigated area (Bavarian Forest National Park) are generally constrained by environmental factors. By contrast, from a local perspective, the two guilds responded similarly to the resource availability gradient. The reproductive traits of the assemblage of saprotrophs were hence influenced by both regional and local environmental filters, whereas ectomycorrhizal fungi were affected only by local filters. Although ectomycorrhizal species in the low productivity area studied did not differ from the expectation from the regional species pool, they clearly responded to resource availability at the local scale. We presently are not able to offer a convincing hypothesis to explain these interesting differences in the importance of regional and local factors between saprotrophic and ectomycorrhizal fungi.

In our study area, elevation is not only correlated with productivity but also with climate. Mean annual temperature decreases with elevation, ranging from 6.5 to 3.5°C in our study area (Bässler 2004). Although we followed the reasonable assumption that resource availability should reflect the response of reproductive syndromes (Carlile et al. 2001a), we are not sure whether temperature or its correlates also contribute to explaining the observed pattern. In this respect, it might be important to consider that fungi are ectothermic organisms with metabolic rates related to temperature (Carlile et al. 2001b). Experiments are needed to disentangle confounding effects between climate variables and resource availability. One further important drawback of our study is that we were not able to estimate the investment into sexual reproduction. This would require information on the belowground biomass of fungal individuals (genet). However, there is no feasible way to obtain such information in ecological field studies across broad gradients (Dahlberg and Stenlid 1994; Guidot et al. 2001, 2004). Whether competitive interactions might also contribute to explain the observed pattern must be left to future studies.

Overall, our study revealed basic differences in the reproductive syndromes between saprotrophic and ectomycorrhizal fungal assemblages. We argue that these differences can be explained in part by their resource acquisition strategies. Furthermore, the results of our study suggest that both regional and local environmental filters affect saprotrophs, whereas only local environmental filters affect ectomycorrhizal fungi. Nevertheless, not all patterns found in our study are consistent with the strategies of resource acquisition. Still, our study provides another aspect of mutualistic relationships. Most studies in this field are from the perspective of the hosts e.g., that demonstrate that plants hosting mycorrhizas are more productive than those without mycorrhiza (e.g., Burgess et al. 1993; Smith and Read 2008; Smith et al. 2010). Our study reversed this perspective, and we show that evolution towards mutualism might have increased the reproductive output of ectomycorrhizal fungi compared to free‐living saprotrophs. However, to deepen our understanding of lifestyle‐specific assemblages, we need to identify the size of fungal individuals (genet) and to estimate the true investment in sexual reproduction along environmental gradients.

## Conflict of Interest

None declared.
